# OCT Guidance in Bifurcation Percutaneous Coronary Intervention

**DOI:** 10.31083/j.rcm2403088

**Published:** 2023-03-08

**Authors:** Athanasios Moulias, Rafail Koros, Angeliki Papageorgiou, Panagiotis Patrinos, Panagiota Spyropoulou, Angeliki Vakka, Maria Bozika, Georgios Vasilagkos, Anastasios Apostolos, Kassiani-Maria Nastouli, Grigorios Tsigkas, Periklis Davlouros

**Affiliations:** ^1^Department of Cardiology, General University Hospital of Patras, 26504 Patras, Greece

**Keywords:** optical coherence tomography, bifurcation lesion, percutaneous coronary intervention

## Abstract

Coronary bifurcation is defined by the European Bifurcation Consensus as a coronary artery stenosis adjacent to the origin of a significant side branch. Its anatomy is composed of 3 different segments: proximal main vessel, distal main vessel and side branch. Coronary artery bifurcation lesions are encountered in approximately 15–20% of all percutaneous coronary interventions and constitute a complex subgroup of lesions characterized by lower procedural success rates and higher rates of adverse outcomes. In recent years, a growing focus in the European and Japanese bifurcation club meetings has been the emerging role of intravascular imaging, in guiding successful bifurcation percutaneous coronary interventions (PCI). In this review we will present the main ways optical coherence tomography (OCT) can be used to improve outcomes during bifurcation PCI.

## 1. Introduction

Coronary bifurcation lesion is defined by the European Bifurcation Consensus as a coronary artery stenosis adjacent to the origin of a significant side branch [1]. Its anatomy is composed of 3 different segments: proximal main vessel (MV), distal MV and side branch (SB) [2]. Coronary artery bifurcation lesions are encountered in approximately 15–20% of all percutaneous coronary interventions (PCI) [3].

**Fig. 1.**
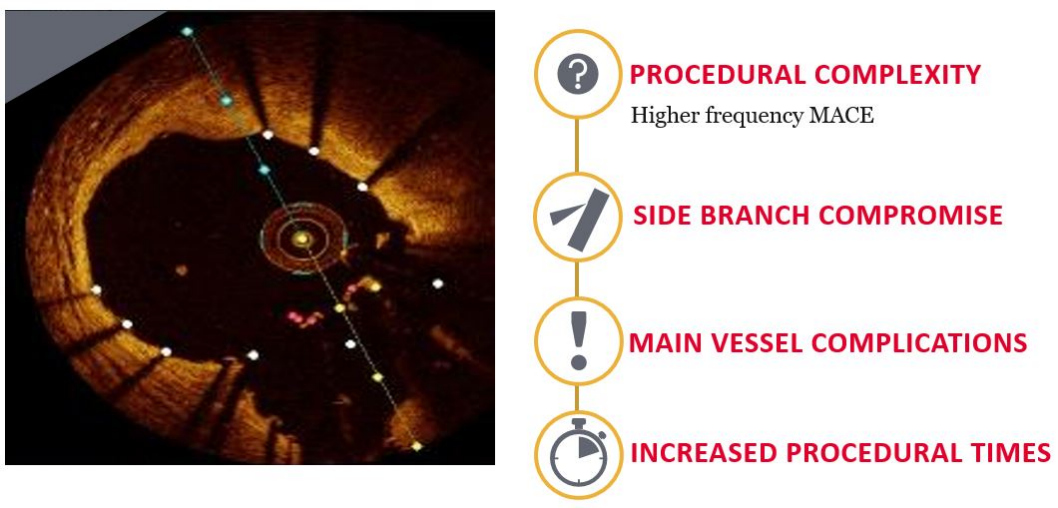
**Impact of bifurcation disease**. Optis is a trademark of Abbott or its related companies. Reproduced with permission of Abbott, © 2022. All rights reserved.

Despite the significant advances in stent technology, bifurcation lesions constitute a complex subgroup of lesions and are characterized by lower procedural success rates and higher rates of adverse outcomes [4] (Fig. 1). Conventional angiography has shown a limited capacity for depicting important features of the complex bifurcation anatomy and periprocedural issues such as the position of the side branch wire. Moreover, conventional angiography has limited value in guiding PCI optimization (stent apposition and expansion) [5]. According to the older COBIS II Registry, SB occlusion occurred in about 8.5% of PCI-treated bifurcation lesions. Intracoronary imaging with optical coherence tomography (OCT) represents a valuable tool for planning and performing bifurcation PCI [6].

## 2. OCT in Percutaneous Coronary Intervention: Rational and Evidence

Intravascular optical coherence tomography (OCT) is a valuable adjunctive tool for guiding coronary bifurcation PCI. OCT provides high resolution (axial 10–20 μm, lateral 20–40 μm) 10 times higher compared with intravascular ultrasound (IVUS) [7]. The OCT catheter is advanced distal to the lesion or stent to be examined, and the pullback is performed with a speed of 10–40 mm/s until either the guiding catheter is reached or the maximal pullback length is completed. During pullback, contrast injection at a rate of 3 mL/s (right coronary) to 4–5 mL/s (left coronary) is required to eliminate red blood cells which scatter the light [8].

OCT provides a clear view of the border between the lumen and the endoluminal lining of the vessel wall. It facilitates the detailed assessment of plaque characteristics and distribution, thereby contributing in the planning of the PCI strategy [9, 10]. It also assists the reliable evaluation of coronary anatomy, lumen area and lesion severity, guiding of SB rewiring and precise detection of stent under-expansion, stent strut malapposition and edge dissection [11]. OPUS-CLASS study proved that OCT provided accurate measurements of coronary lumen with excellent intraobserver reproducibility compared with quantitative coronary angiography (QCA) and IVUS whereas OCT was much more sensitive in detecting suboptimal PCI result compared with IVUS [12]. Co-registration of OCT and angiography in complex bifurcations provides the advantage of reducing the risk of overlap and foreshortening [10]. The DOCTORS study showed that without access to the co-registered landing zone, parts of the OCT-identified lesion area to be covered by stent were left uncovered in 70% of the investigated lesions [13].

The superior resolution of OCT provides potential advantages over IVUS for specific steps of bifurcation interventions, including visualization of the site of guidewire crossing and stent optimisation tools [14, 15]. Furthermore, it presents greater sensitivity for detection of stent-related problems (dissection, malapposition, thrombus or tissue protrusion) [14]. According to an imaging substudy of the OPINION trial, immediately after PCI, OCT-guided PCI was associated with a trend for smaller minimum stent area, fewer proximal stent-edge hematomas, and fewer irregular protrusions than IVUS-guided PCI. At 8 months, the neointima area tended to be smaller in the OCT-guided PCI group than in the IVUS-guided PCI group, although the percentage of uncovered struts was significantly higher in the OCT-guided PCI group than in the IVUS-guided PCI group [16]. The ILUMIEN III study was a controlled, randomized trial which compared OCT-guided, IVUS-guided, and angiography-guided PCI. OCT-guided PCI patients were treated according to an algorithm based on measurement of the external elastic lamina in the proximal and distal reference segments, designed to achieve larger stent dimensions and more complete lesion coverage than would occur with sizing to the distal and proximal reference lumens. OCT-guided PCI resulted in significantly greater minimum and mean stent expansion compared with angiography-guided PCI. The trial showed non-inferiority of OCT-guided PCI to IVUS-guided PCI in terms of minimum stent area (5.79 ${\mathrm{mm}}^{2}$ vs 5.89 ${\mathrm{mm}}^{2}$). However, OCT guidance resulted in fewer untreated major dissections than IVUS guidance (14% vs 26%) and fewer areas of major stent malapposition than both IVUS guidance and angiography (11% vs 21% vs 31%). Finally, the reference segment external elastic lamina-based OCT stent sizing strategy was safe, with few procedural and 30-day major adverse events, which were comparable between groups [17].

The development of three dimensional (3D) OCT allows a better evaluation of coronary anatomy and facilitates recognition of the anatomical changes after intervention compared with two dimensional (2D) OCT [11]. The application of 3D reconstruction creates a volume of the location of interest from the OCT pullback, overcoming the limitations of the 2D methods. This technology has recently become widely available (3D bifurcation mode, Optis™ Stent Optimization Software, St. Jude Medical, St. Paul, MN, USA) and automatically recognises the carina as well as side branch ostia with a diameter of ≥1.5 mm [10]. “Stent roadmap” shows the position of the minimal lumen area and suggests the proximal and distal landing zones for the stent placement based on the mean lumen area measurements. Furthermore, reconstruction from automatic lumen delineation allows a superimposition of malapposed struts [10]. The Medis company (Leiden, the Netherlands) 3D OCT software system has developed 3D angiography and OCT co-registration which enables quantitative assessment of the side branch ostium by using an OCT pullback from the main branch (MB) in a cross-section which is perpendicular to the side branch centreline. For precise sizing of a side branch ostium, OCT pullbacks could be performed in both the MV and SB, which is often challenging in clinical practice due to safety concerns including the use of an excessive amount of contrast media [10]. The multicentre OPTIMUM clinical trial enrolled patients with angiographically significant bifurcation lesions treated with provisional stenting strategy using drug eluting stent. One group of patients underwent 3D-OCT assessment after rewiring into the jailed side branch after stenting and proximal optimisation technique, while the other group underwent conventional angiographic guidance. The study proved superiority of 3D-OCT-guided PCI compared with the angiography-guided PCI in the terms of malapposed stents per lesion. The average percentage of incomplete stent apposition per lesion at bifurcation was lower in the 3D-OCT guidance arm than that in the angiography guidance arm (19.5 ± 15.8% vs 27.5 ± 14.2%, *p* = 0.008). The feasibility of the online 3D-OCT system was 98.2% in contrast to 89.9% in the older Murasato *et al*. [18] study using offline 3D OCT system [19]. Moreover, 3D OCT reconstruction of coronary bifurcation enables computational flow dynamics, simulation of flow velocity and pressure (fractional flow reserve) [18].

## 3. Evaluation of Coronary Bifurcation Lesion Anatomy with OCT

**Fig. 2.**
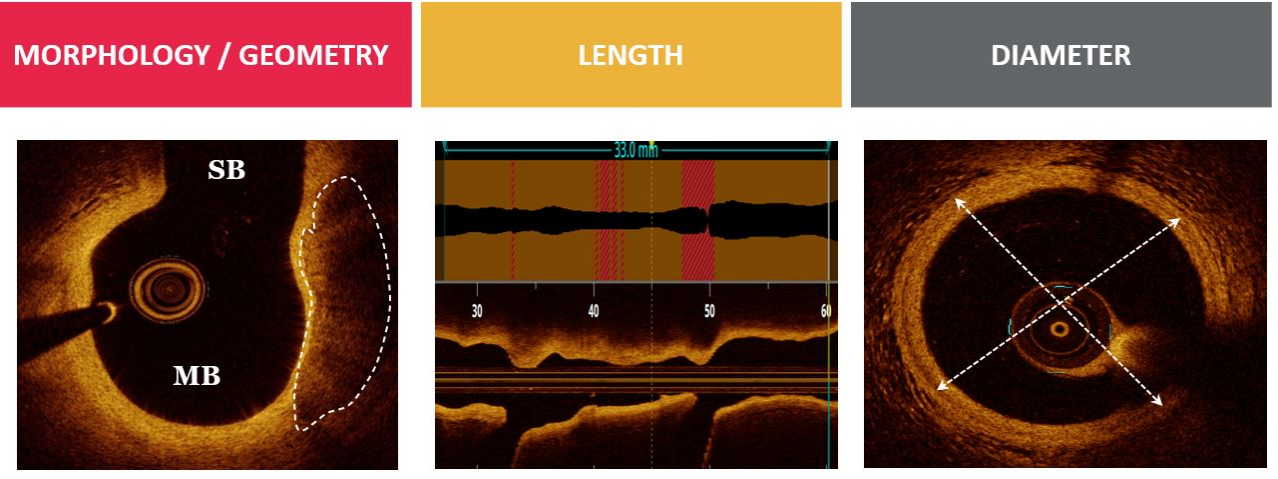
**Evaluation of coronary bifurcation lesion anatomy with OCT**. Optis is a trademark of Abbott or its related companies. Reproduced with permission of Abbott, © 2022. All rights reserved.

As previously referred, OCT provides significant preprocedural assessment of the atherosclerotique plaque composition and morphology, lesion length and diameter, as well as bifurcation geometry (Fig. 2). OCT is helpful for evaluation of the shaft and distal part of the left main artery, although aorto-ostial imaging is not feasible [20]. Adequate flushing of the vessel is required for the careful assessment of its anatomy [11]. It is important to ensure that the maximum scan range is displayed on screen and increased flow of flushing agent is used [21].

Atherosclerotic plaque and its composition play a key role when assessing the risk of SB compromisation following MV intervention [10]. Atherosclerotic lesions tend to form at specific regions with low shear stress. Coronary plaque is present predominantly in the region opposite the flow divider, whereas the flow divider (the region of high wall shear stress) is rarely affected. OCT can assess the circumferential extension and the depth (superficial vs deep) of calcification [22]. The depiction of extensive calcification on OCT is associated with suboptimal stent expansion, stent malapposition, and failure of device delivery.

OCT acquisition in the SB might be valuable in bifurcation lesions with large SBs as it can contribute to the selection of the most appropriate PCI strategy. During MV pullback, it is important to evaluate if the side branch ostium is visible. In case the shadow of the guidewire obscures the ostium of the SB, manipulation of the guidewire and repeating pullback is necessary to obtain the appropriate information [21]. Care must be taken when the stiff OCT catheter is advanced after predilatation of the SB into an angulated SB, due to the increased risk of worsening a dissection previously caused by the predilatation procedure. It is not recommended to cross a jailed SB as this might cause OCT catheter entrapment and distortion of the MV. Measurement of the SB ostium can be obtained in a cross-section with a well visible SB at the carina point. However, there is a risk of missing smaller areas in non-perpendicular planes, as this method is highly dependent on the angulation of SB [23]. The ostium of the SB can also be measured by utilizing multiple cross-sectional views. One method of this type assesses the area of the oval opening of the SB by counting the number of cross-sections where the SB is visibly multiplied by the thickness of cross-sections multiplied by the diameter of the largest opening of the SB multiplied by ¼*π. The disadvantage of this method is that the ostium area is non-perpendicular to the SB, again resulting in overestimation of the largest diameter and area of the ostium. Another method assesses the ostial area by measuring the SB width in all cross-sectional views with a visible SB. Subsequently, the width measurements are added together and multiplied by the distance between the cross-sections. Although time consuming, the advantage of this method is the perpendicular assessment of the SB ostium in relation to the MV [23].

**Fig. 3.**
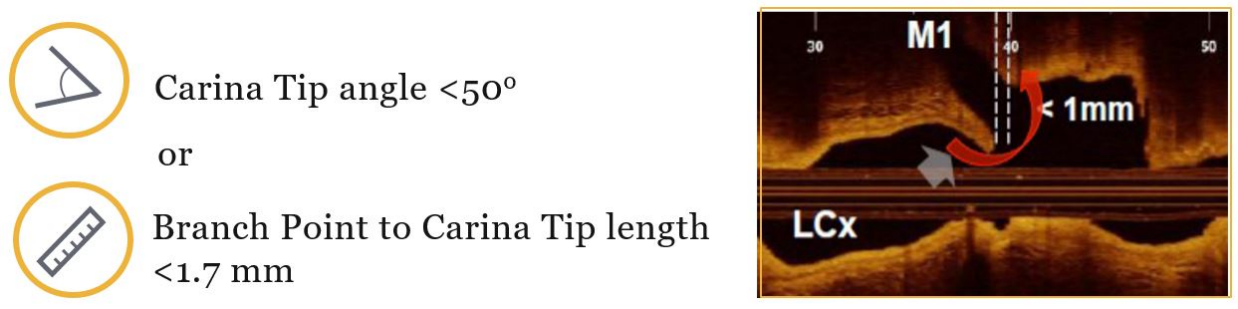
**Predictors of side branch complications**. Optis is a trademark of Abbott or its related companies. Reproduced with permission of Abbott, © 2022. All rights reserved.

OCT acquisition of SB describes the anatomic characteristics which constitute angiographic predictors for SB occlusion. The OCT study by Watanabe *et al*. [24] demonstrated that a carina tip angle less than 50° and a branching point-carina tip length less than 1.70 mm were predictors of side branch compromise after MV stent implantation (Fig. 3).

## 4. Stenting in Bifurcation Lesion Using OCT Guidance

**Fig. 4.**
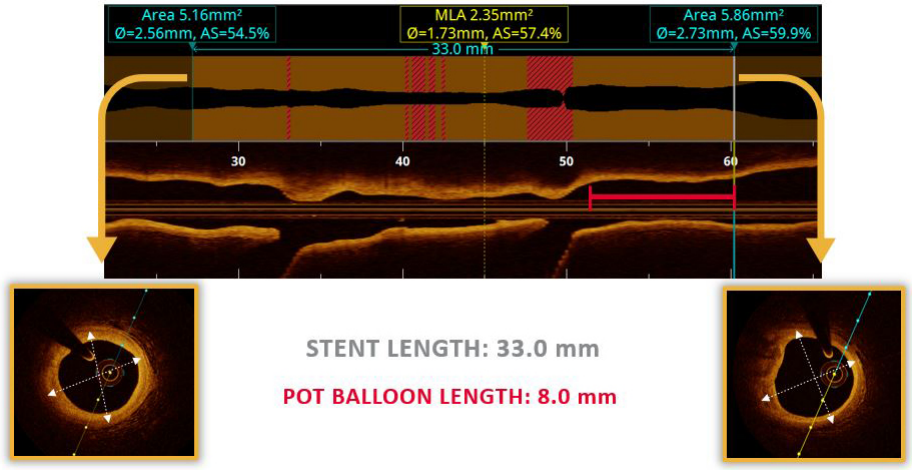
**Stent sizing based on OCT measurements**. Optis is a trademark of Abbott or its related companies. Reproduced with permission of Abbott, © 2022. All rights reserved.

OCT can play a fundamental role in choosing the appropriate size of the stent which should be implanted and positioning of the stent. OCT is very useful in the estimation of the proximal and distal reference segments in the minimally diseased vessel areas adjacent to the bifurcation lesion (Fig. 4). The vessel size is estimated by contouring the media layer in the respective cross-sectional view. Sizing of the vessel can be operated by OCT guidance using either external elastic membrane areas or lumen areas. Whenever the proximal vessel is too large for the external elastic membrane (EEM) to be measured, stent sizing according to lumen area measurement is recommended. The size of the stent should be selected aiming at the fractal geometry of bifurcation according to the law of flow conservation. The MV stent should be sized according to the distal MV reference diameter, whereas the MV stent should allow for expansion to the reference diameter of the proximal MV. It is necessary to cover the bifurcation stenosis segment at least 6–8 mm from the proximal stent edge to the carina, to enable the appropriate proximal optimisation technique (POT) with the shortest balloon. Appropriate stent sizing is determinant factor in bifurcation lesions due to the fact that stent oversizing in the MV can cause carina shifting, thereby inducing SB distortion and narrowing. POT results in better stent struts’ apposition in the proximal MV, facilitates SB wiring, reduces the risk of abluminal rewiring, and lowers the risk of catheter-induced stent distortion during the procedure [25, 26].

Two fundamental treatment approaches for bifurcation lesions have been broadly used: provisional stenting and two-stenting approach. Provisional stenting is the most frequently used interventional treatment in bifurcation lesions. It is conducted by stent implantation in the MV, followed by post-dilatation of the stent at the level of the proximal MV with a balloon diameter sized 1:1 according to the proximal MV (POT). SB dilatation should be considered before MV stenting in complex bifurcation lesions in which the lesion is very severe, angulated or highly calcified. Proceeding to SB stenting is performed only if its angiographic appearance after MB stenting is considered suboptimal [27].

Two stent-approach is the preferred approach for complex bifurcation lesions involving large and diseased SB. Final kissing balloon inflation is regarded as mandatory step, and failure to adequately perform it, has been associated with adverse clinical outcome [10, 27].

**Fig. 5.**
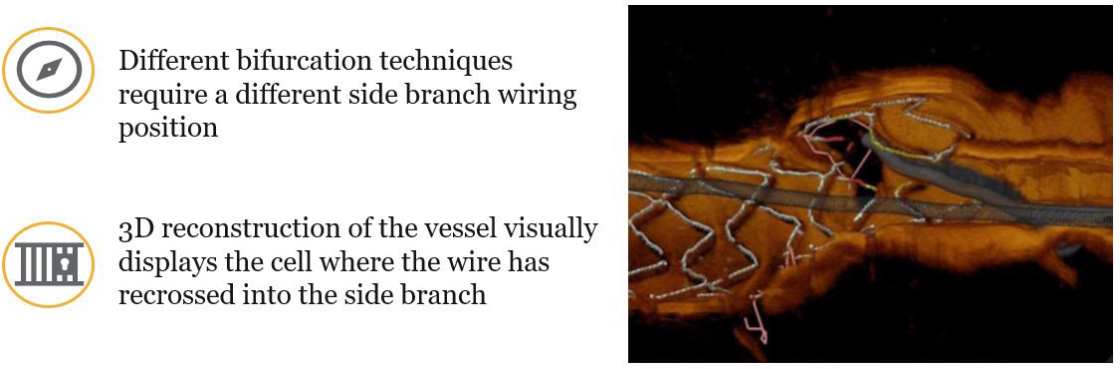
**OCT-guided side branch rewiring**. Optis is a trademark of Abbott or its related companies. Reproduced with permission of Abbott, © 2022. All rights reserved.

Wire recrossing to the SB is needed, when upfront two-stent appoach is chosen or in provisional stenting, when there is impairment of SB flow after stenting the MV. A distal stent cell position for recrossing reduces the extent of the metallic carina and achieves adequate stent expansion and stent struts’ apposition at the ostium of the SB. It should be noticed that a very distal strut position might increase the risk of abluminal rewiring of the SB stent and subsequently SB dilatation will crush the SB stent. OCT is considered as a helpful tool to guide rewiring in provisional and two-stent strategy by recognizing accidental abluminal rewiring and assessing the position of the recrossing wire (Fig. 5). Alegria-Barrero *et al*. [28] have shown a significant reduction of malapposed stent struts in patients undergoing elective treatment of bifurcation lesions using provisional stenting strategy and OCT guidance.

OCT has been widely used to evaluate the procedural result of new bifurcation stenting technologies. Most case reports visualise the complex anatomical structure of bifurcation dedicated stents in 3D OCT. Ferrante *et al*. [29] used OCT to assess the efficacy of the Tryton dedicated side branch stent in nine patients, and found that malapposed stent struts were more frequently seen at the level of the bifurcation than in the proximal and distal stent in the MV. In particular, the highest proportion of malapposed struts was seen towards the ostium of the side branch [29, 30]. The following stents are used with provisional SB stenting approach. These stents have been arranged into 3 categories: self-alignment devices (SLK View™ [Advanced Stent Technologies, Pleasanton, CA, USA], Frontier™ [Guidant Corporation, Santa Clara, CA, USA], Twin Rail™ [Invatec/Medtronic, Roncadelle, Italy], Nile Croco® [Minvasys, Gennevilliers, France], Petal™ [Boston Scientific, Natick, MA, USA] and Abbott SBA [Abbott Vascular, Redwood City, CA, USA]), controlled-alignment stents (Trireme and Side-kick) which require less wire wrap, but three guidewires have to be inserted, and no alignment required stents (Stentys). Self-alignment stents require the insertion of two non-twisted wires, optimal predilatation of both branches, twisted wires, which must be corrected before further advancement by withdrawal and re-positioning of one of the wires, and anticipation of inadequate rotation requiring better preparation of the lesion. Implantation of the STENTYS® (Self-Apposing® stent; Stentys S.A., Paris, France) stent, previously coated with Paclitaxel and now with Sirolimus, is achieved by self-deployment of the stent by inflation of a balloon breaking an external membrane. A guidewire is inserted into the SB through the stent struts. Balloon inflation enables the connections between struts to be broken, resulting in the stent struts being pushed into the SB ostium. A second drug-eluting stent (DES) may be implanted in the SB as required. The Sideguard Capella, a conical, self-expandable, eluting stent which may be difficult to position at the ostium and the Tryton Side Branch stent dedicated to SB stenting, are equipped with an anchoring system for implantation in the proximal main vessel (PMV). Both stents can be used as a single stent. However, they are designed to be deployed in the PMV in a T stenting and Culotte configuration respectively. Of these two stents, the most thoroughly assessed so far has been the Tryton Stent [10].

Bioresorbable scaffolds (BRS) represent a promising novel technology that theoretically can eliminate the risk of late and very late stent thrombosis observed after deployment of DES. It constitutes a treatment approach for coronary narrowing which provides transient vessel support with drug delivery capability. The most widely studied BRS to date is Absorb™ [Abbott Vascular, Redwood City, CA, USA], Magmaris™ [Biotronik, Bülach, Switzerland], DESolve ® [Elixir Medical Corporation, Milpitas, CA, USA], Fantom® [REVA Medical, San Diego, CA, USA] and ART [Arterial Remodeling Technologies, Paris, France], although the Absorb scaffold has been withdrawn from the market by the manufacturer. The poly-lactide or magnesium mechanical properties of the bioresorbable materials are weaker than those of permanent metals. The struts are thicker and wider and the large crossing profile of the delivery system is characterized by increased thrombogenicity. These limitations with the use of complex techniques or even with final kissing balloon (FKB) may cause damage to the MB stent and warrant their discouraged use [31, 32]. The European Bifurcation Club has recently recommended regarding BRS use in bifurcation lesions the “mini-kissing balloon” technique, with minimal overlap of the balloons. It is crucial to select the MB stent diameter according to the MB distal reference, knowing the limitations of post stent deployment further dilatation. The stent should be deployed slowly (2 atmospheres every 5 seconds) and the POT technique should be used to appose the proximal part of the MB stent. If the SB is compromised, the strut should preferably be opened toward the SB with a non-compliant (NC) balloon (≤2.5 mm), followed by POT with a larger balloon in the proximal MV to correct scaffold malapposition. OCT is necessary to diagnose acute disruptions and late discontinuities of the polymeric BRS, due to the fact that these complications remain undetectable by angiography and/or IVUS. 3D OCT facilitates the classification of the jailed side branch according to the number of compartments created by the criss-cross of the struts as well as the configuration of the jailing struts [10, 27].

## 5. Postprocedural OCT Evaluation

**Fig. 6.**
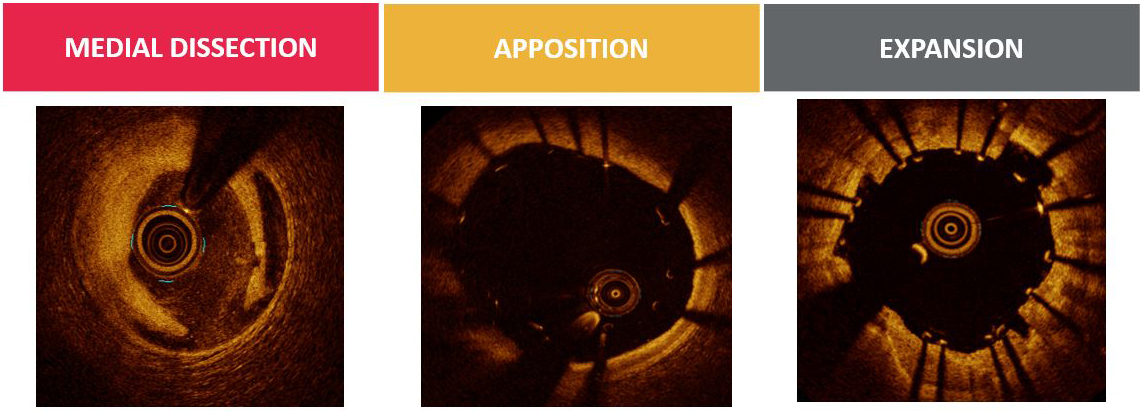
**OCT assessment of PCI outcome**. Optis is a trademark of Abbott or its related companies. Reproduced with permission of Abbott, © 2022. All rights reserved.

Following revascularization in a bifurcation lesion using provisional or two-stent strategy, OCT is of great value in PCI optimization, in terms of recognition of stent underexpansion, struts’ malapposition, in-stent tissue protrusion, edge dissection, geographic miss and in-stent thrombus (Fig. 6).

**Fig. 7.**
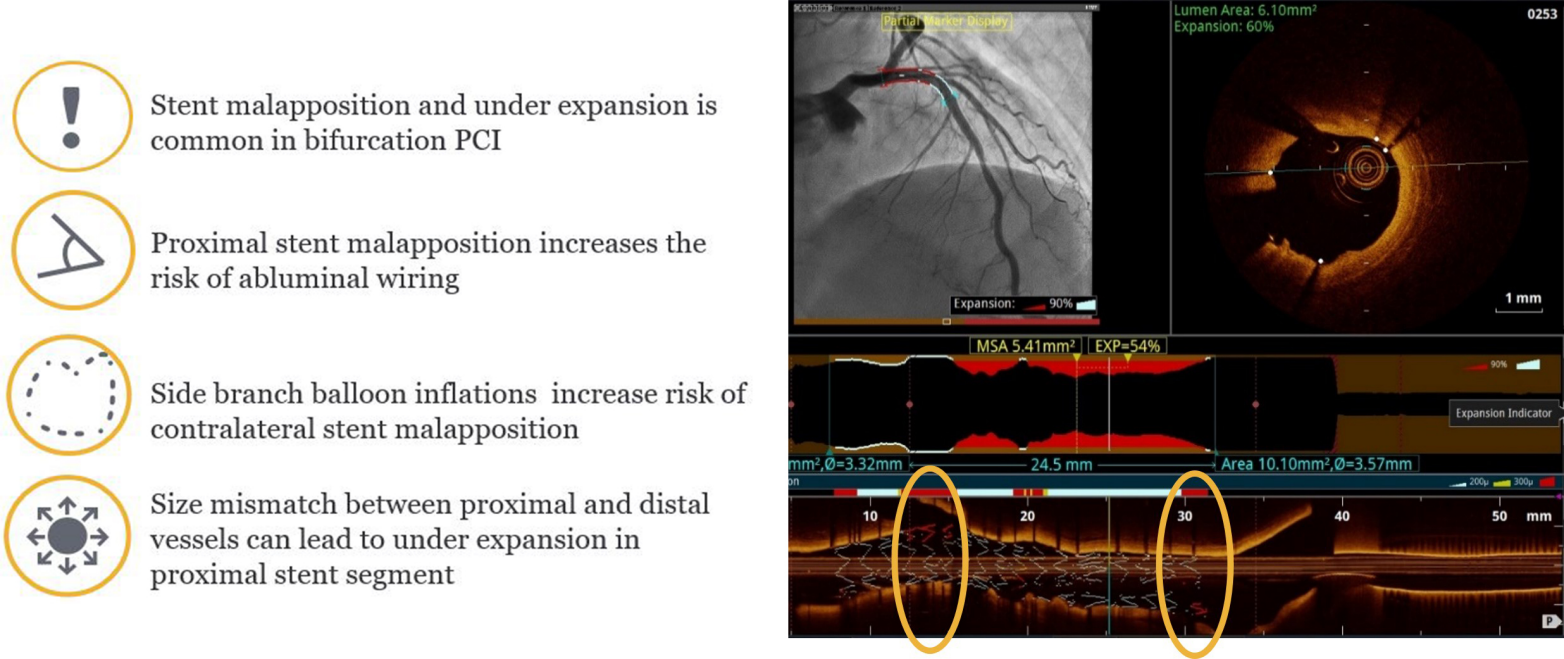
**OCT assessment of bifurcation apposition**. Optis is a trademark of Abbott or its related companies. Reproduced with permission of Abbott, © 2022. All rights reserved.

In bifurcation lesions stent underexpansion is associated with adverse clinical outcomes. Stent under-expansion is defined as an in-stent minimum lumen area <70% of the average reference lumen area (according to Prati *et al*. [33]) or as a minimal stent area of the proximal and/or distal segment <90% of the proximal and/or distal reference lumen area respectively (according to ILUMIEN III trial). Recently, a refined OCT-based parameter of stent expansion, the minimum expansion index, that yields the ideal lumen area in each frame by taking into account vessel tapering, has been shown to correlate with device-oriented clinical endpoints [34]. Stent expansion should be evaluated separately in the proximal MV, distal MV and SB, with respect to each reference area. OCT provides the advantage of selection of an appropriate balloon for post dilatation in order to achieve full expansion (Fig. 7) [11, 17, 35].

Stent malapposition is more common at the proximal MV and tissue prolapse or dissection at the distal MV segment. Acute strut malapposition could persist (persistent malapposition) leading to higher rate of long-term major adverse cardiovascular events (MACE) or resolve at follow-up (resolved malapposition) with no clinical impact, whereas strut malapposition could also develop during follow-up (late acquired malapposition). Malapposition in which the distance from the endoluminal lining of the strut to the vessel wall is <250 μm, is more likely to have recovered at follow-up, and not need any additional correction [35, 36].

The edge dissections’ severity is defined by the presence of the following factors in OCT acquisition: the longitudinal (≥3 mm) and circumferential extension (≥60 degrees) of the dissection, the intra-dissection lumen area respective to the reference (<90%) and the depth of the dissection (media or even adventitia) or flap thickness [33, 37]. The thickness may also be a practical indicator of significant stent edge dissection among several anatomical metrics.

Geographical miss is another PCI complication possibly observed during post procedural OCT assessment of bifurcation lesion. OCT guidance and subsequent stenting are indicated in untreated minimum lumen area (MLA) ≤60% of adjacent reference segment lumen area up to 10 mm from the proximal and/or distal stent edges [38, 39].

OCT has significant diagnostic value in the assessment of cases of stent failure in bifurcation lesions including stent thrombosis, in-stent restenosis and neointimal hyperplasia. The main cause that leads to stent thrombosis is the presence of stent struts at the core bifurcation segment due to jailing of struts or non-apposed struts. Secondly, another important cause is the presence of compromised stented side branch because of underexpansion at the ostium of the SB, or because of an accumulation of several layers of stent struts leading to a delayed healing process leaving uncovered struts which are more prone to thrombus formation. Furthermore, compromise of the non-stented SB, usually due to plaque shift or carina shift or plaque overgrowth and problems remote from the core bifurcation segment, but related to the specific character of bifurcation PCI such as problems due to double or triple layers of struts in the proximal main vessel or problems due to more extensive manipulation of stents in bifurcation PCI have also been implicated in stent thrombosis. Cases of early stent thrombosis could also be attributed to ineffective platelet inhibition or due to systemic disease like cancer or major infection. In cases where malapposition, underexpansion or uncovered struts have been identified as the most possible cause of stent thrombosis, corrective measures with thrombus aspiration and/or additional balloon dilatation are usually sufficient to ensure a good final result [10]. 3D OCT can be used to detect scaffold disruption in radiolucent bioresorbable scaffolds and may lead to the use of fewer additional stents in the treatment of stent thrombosis. When excessive neointimal hyperplasia or in-stent neoatherosclerosis is considered as the dominant cause of stent failure, OCT may be used to guide lesion preparation [10, 21].

## 6. Conclusions

The treatment of bifurcation lesions has remained one of the most challenging issues in interventional cardiology in spite of the advances in stent technology and carries a higher incidence of target lesion failure than other forms of PCI. The consensus is that main branch stenting with provisional SB stenting should be the default approach in the majority of cases. OCT is the intracoronary imaging modality with the highest resolution and can generate automatically contoured lumen areas across the variable geometry of bifurcation lesions. Therefore, OCT may play an important role in understanding bifurcation geometry and be used to predict side branch complications. Lesion morphology, length, vessel diameter, edge complications, strut malapposition and stent expansion can all be accurately assessed using OCT during bifurcation PCI. OCT guided side branch rewiring may lead to optimal positioning and reduced strut protrusion compared to angiography-based guidance. New technological advances hold the promise of improved design of OCT catheters, facilitating imaging even in the most challenging anatomy.
